# Medium-Term Outcomes from a Series of 1000 One Anastomosis Gastric Bypass in Australia: A Case Series

**DOI:** 10.1007/s11695-024-07213-5

**Published:** 2024-04-12

**Authors:** Laura Hailstone, David Tovmassian, Chu Luan Nguyen, Pearl Wong, Philip Alexander Le Page, David Martin, Craig Taylor

**Affiliations:** 1 Upper Gastrointestinal Surgery, Concord Repatriation General Hospital, Sydney, NSW Australia; 2 University of Sydney, Sydney, NSW Australia

**Keywords:** One anastomosis gastric bypass, Bile reflux, Mini gastric bypass, Metabolic surgery Australia

## Abstract

**Purpose:**

This study presents the short- (less than 6 months) and medium-term (6 months to 2 years) outcomes for weight loss and type 2 diabetes mellitus (T2DM) for all patients undergoing one anastomosis gastric bypass (OAGB) across multiple institutions between 2015 and 2021.

**Materials and Methods:**

A retrospective analysis of prospectively collected databases was performed including 1022 participants who underwent OAGB at multiple institutions by multiple surgeons between 2015 and 2021. Primary outcome was percentage total weight loss (TWL) and secondary outcomes were achieving resolution of T2DM; OAGB specific short- and medium-term complications including bile reflux, marginal ulceration and internal herniation.

**Results:**

One thousand and twenty-two patients underwent OAGB (81% primary surgery). A percentage of 34.1% (*n* = 349) had a preoperative diagnosis of type 2 diabetes mellitus (T2DM). Mean TWL was 33.6 ± 9%  with a T2DM remission rate of 74% at 1-year post-op. Rates of bile reflux and marginal ulceration was 1.1% (*n* = 11) and 1.1% (*n* = 11). There were no cases of internal herniation during the follow-up period.

**Conclusion:**

OAGB results has echoed previously published work as being efficacious and safe in a short-medium term. The prevalence of complications, especially bile reflux is overall low in our population and no current evidence exists to support an increased risk of metaplasia or malignancy related to bile within the stomach.

**Graphical Abstract:**

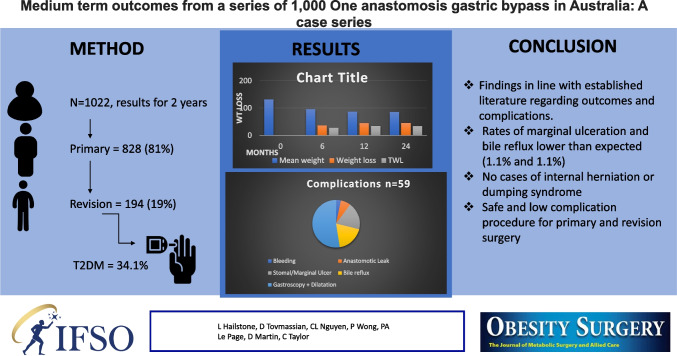

## Introduction

Obesity continues as a global, largely preventable epidemic which affects over a third of people worldwide [1]. Multiple associated medical problems impact upon the quality and quantity of life of individuals. Medical management has so far shown only modest improvement in obesity and related medical problems [2, 3]. Bariatric metabolic surgery has shown promising results due to improved short-term outcomes associated with laparoscopic approaches obtaining durable weight loss and associated medical problem control when compared with medical management alone [3]. Restrictive alone or combined restrictive/malabsorptive procedures are the mainstay of surgical management of obesity with many procedures being pioneered in the recent past.

As bariatric metabolic surgery is a rapidly growing and changing field, it remains prudent to continually compare surgical techniques available to ensure not only appropriate weight loss and associated medical problem control but also safety of these procedures. One anastomosis gastric bypass (OAGB) is a procedure in the armamentarium of bariatric metabolic surgeons which is not without controversy. OAGB may also be referred to as either single anastomosis gastric bypass, omega loop gastric bypass or mini-gastric bypass, and multiple centres have published promising results with respect to weight loss and complications utilising this procedure [4, 5]. OAGB is the second most common primary bariatric metabolic procedure performed in New South Wales, Australia. The procedure consists of a modified Mason’s loop gastroenterostomy with a linear gastric pouch fashioned along the lesser curve and a single anastomosis gastroenterostomy providing combined restrictive and malabsorptive facets to achieve weight loss. Proponents of the procedure favour the reduced complication profile whilst maintaining non-inferior weight loss and associated medical problem outcomes when compared with Roux-en-Y gastric bypass (RYGB) [5]. Critics of the procedure have concern that bilious reflux can lead to symptomatic reflux disease and potentially related sequelae such as metaplasia and malignancy [6].

This case series presents the short- to medium-term outcomes of all patients undergoing OAGB across multiple institutes by multiple surgeons either as a primary operation or as a revision procedure. We hypothesise that our results will be in line with published literature on OAGB with regards to weight loss and associated medical problem control with possibly lower bile reflux.

## Methods

A retrospective analysis of a prospectively collected data base was conducted on 1022 patients who underwent an OAGB between 2015 and 2021. Operations were performed by three surgeons across three hospitals in Sydney, Australia. De-identified data was extracted from surgical practice databases by two independent authors. Inclusion criteria included any patient who underwent primary or revisional OAGB within the study period. Exclusion criteria was patients who did not attend at least one follow-up appointment.

Baseline data included age, gender, weight, body mass index (BMI) and biochemical T2DM status (HbA1c). This data was recorded in the surgeon’s initial consultation for primary surgery or at the time of decision to undergo revisional surgery. Post-operative weight and complications were routinely recorded at 6 months, 1 year and 2 years’ time points. Instances whereby a significant complication occurred within the routine follow-up period but outside these time points and made known to us have been recorded for transparency. Outcomes were included until the time of most recent follow-up. The primary outcome was percentage total weight loss (TWL), defined as weight loss achieved divided by highest recorded weight prior to surgery. Secondary outcomes included presence surgery related complications—specifically bleeding, early return to operating room, anastomotic leak, strictures/stenoses, bile reflux, marginal/stomal ulceration and internal herniation with reference to the Clavien-Dindo classification [7]. Secondary outcome also included biochemical remission of T2DM defined as HbA1c < 6.0%.

In line with bariatric metabolic surgery guidelines at the time of patient care during the study, criteria for surgery used was BMI > 35, or BMI > 30 with obesity-related medical problems as per NIH guidelines from 1991 [8]. This paper was primarily aimed at assessing the short-medium term outcomes for patients undergoing OAGB. The authors define short term as up to 6 months post-operative and medium term as a minimum of 6 months up to 2 years post-procedure.

Pre-operative preparation included a multidisciplinary assessment by a bariatric metabolic surgeon, a dietician and a psychologist. Additional preparation included 2 to 4 weeks of a very low-calorie diet to reduce fatty liver bulk and visceral adiposity prior to surgery.

Surgical technique was standardised and was performed in the supine position with a Nathanson liver retractor in the epigastrium, 3 × 12 mm and 1 × 5 mm laparoscopic trocars. A stapled, thin gastric tube of approximately 15 cm in length is fashioned along the lesser curve with the guidance of a 36F bougie. Bilio-pancreatic limb length varied from 150 to 250 cm depending upon surgeon and patient factors. Gastroenterostomy was performed with either completely hand-sewn technique or hybrid stapled with oversewing of the common enterotomy.

Post-operatively, patients were followed up by a multidisciplinary team including a bariatric physician, dietician and psychologist every 3 months in the first year, six monthly in the second year and then annually thereafter. Serum biochemical markers were assessed at 6-month intervals with prescription of additional supplementation when indicated. All patients were prescribed specific multivitamins daily and a proton pump inhibitor (PPI) daily for a minimum of 12 months post-operation.

For statistical analysis, we used descriptive statistics. All data were first tested for normality using the Kolmogorov–Smirnov and Shapiro–Wilk tests. Categorical variables were expressed as *n* (%). Continuous normally distributed variables were expressed with their means and standard deviations, while non-normally distributed variables were expressed with their medians and interquartile ranges. All statistical tests were conducted using IBM SPSS statistics program version 26 (IBM SPSS Statistics, Version 2.0. Armonk, NY: IBM Corp.) at a ≤ 0.05 significance level.

This case series has been reported in line with the PROCESS Guideline [9].

## Results

From 2015 to 2021, 1022 patients underwent an OAGB, patient demographics are presented in Table 1. Of these patients, 828 (81%) underwent a primary OAGB and 194 (19%) underwent revisional OAGB; after LSG (*n* = 71), LAGB (*n* = 106), previous LAGB to LSG (*n* = 15) or gastric balloon (*n* = 2). The majority of patients were female (71%) and mean age was 43 ± 12. Mean pre-op BMI was 46.7 ± 8.8. Pre-operatively, 349 (34.1%) patients had a current diagnosis of T2DM.

**Table 1:** Baseline demographics

Patient variables	
Patients, *N*	1022
Age, mean ± SD	43.3 ± 11.9
Male, *N* (%)	297 (29.1%)
Primary surgery, *N* (%)	828 (81%)
Preoperative weight, mean ± SD	131.2 ± 29.2 kg
BMI, mean ± SD	46.7 ± 8.8
Excess weight, mean ± SD	60.9 ± 26 kg
T2DM, *N* (%)	349 (34.1%)
OSA, *N* (%)	301 (29.4%)
Hypertension, *N* (%)	387 (37.9%)

Table 2 outlines weight loss outcomes and complications over the follow-up period for all patients. At a 1-year post-OAGB, mean weight loss was 44.6 ± 17.4 kg equating to 33.6 ± 9% mean TWL for all patients. Of the patients undergoing primary surgery, at a 2-year post-OAGB, the mean weight loss was 46.4 ± 19.5 kg with mean TWL 34.6 ± 9.4%. In comparison to patients undergoing revisional surgery, at a 2-year post-OAGB, the mean weight loss was 33.9 ± 14.9 kg with mean TWL 25.7 ± 8.7%.

**Table 2:** Weight loss outcomes and complications over time

Time		0	6 months	1 year	2 years
Mean body weight (kg)		131.2 ± 29.2	95.3 ± 20.9	86.2 ± 19.8	85.2 ± 18.2
Mean weight loss (kg)			35.8 ± 12.1	44.6 ± 17.4	44.8 ± 19.3
Mean percentage TWL (%)			26.9 ± 6.7%	33.6 ± 9.0%	33.4 ± 9.8%
Complications (*N*, %)					
Bleeding	2 (0.2%)				
Anastomotic leak	4 (0.4%)				
Stomal/marginal ulcer	11 (1.1%)				
Bile reflux	11 (1.1%)				
Gastroscopy + dilatation	31 (3%)				

The incidence of T2DM pre-operatively was 34.1%. Amongst these patients, mean HbA1c was 8.3 ± 1.4%. This improved to a mean HbA1c of 5.9 ± 1.05% at a 1-year post-surgery for these patients which equates to a biochemical remission of 74% (*n* = 34).

Of the 1022 patients enrolled in the study, there were a total of 62 (6.1%) patients with a Clavien-Dindo III post-operative complication. There were no deaths during the study period. Four patients had anastomotic leaks (0.3%); one was converted to RYGB on day 6, one required percutaneous drainage on day 4 and one required endoscopic pigtail drain at 9 weeks. Six other patients were returned to theatre (0.6%); one for washout of a deep surgical site infection not related to anastomotic leak, three for an obstructed BP limb, one for small bowel obstruction due to herniated small bowel through a laparoscopic port site on day 8 and one for small bowel obstruction due to adhesions at 2 months. There were 11 stomal ulcers reported within the study period (1.1%); one with perforation at 6 months requiring omental patch repair, one with perforation at 2 years requiring RYGB conversion, one with a bleed treated with endoscopic injection of adrenaline and one with distortion of biliary limb requiring laparoscopy and 6 treated medically. There were 11 (1.1%) cases of bile reflux within the study period all of which were converted to RYGB. Thirty-one patients underwent gastroscopy with dilatation (G&D) for anastomotic stenosis (3%).

## Discussion

OAGB remains a controversial procedure with many surgeons preferring to perform RYGB as their preferred primary bariatric procedure when considering an intestinal diversion option [10]. Our unit having adopted OAGB and performing high volume has shown that our data is in concordance with the hypothesis in published literature that OAGB appears to be equivalent to RYGB with respect to weight loss, associated medical problems control and possible superior for short-medium term complications [4, 5, 11]. Although not all associated medical problems have been presented, we achieved a diabetes resolution of 87.4% at 1 year which is in line with published intestinal diversion procedures [3].

The major feared complication of OAGB by detractors of the procedure remains bile reflux. Only 1.1% of our cohort had medically refractory bile reflux requiring conversion to RYGB; this is in stark contrast to views that exist that bile reflux is a prevalent complication in this patient cohort [12]. In addition to this, we have yet to identify a case of metaplasia either in the gastric pouch or the oesophagus in our OAGB patients. Despite tens of thousands of patients having OAGB worldwide over more than two decades, there remains no published evidence linking OAGB to malignancy. This theory seems to stem from data of patients who had previously undergone Billroth-II reconstruction following distal/subtotal gastrectomy. Significant confounders exist to accurately extrapolate those findings to patients undergoing bariatric surgery [13].

Stomal ulceration in our cohort appears in 1.2% of our cohort which is below the reported rate for intestinal diversion procedures, published randomised data shows that stomal ulceration rate of OAGB compared to RYGB is similar at approximately 3–5% [5, 14]. Our populations lower risk may be due to study design limitations and we cannot make definitive conclusions without an appropriate comparator group; however, we do exercise a higher degree of caution for those undergoing OAGB who are smoking often times offering a different procedure. We also provide routine extended duration post operative PPI prophylaxis and strong education on the importance of avoiding non-steroidal anti-inflammatory medications.

The complication profile in our population undergoing OAGB with respect to internal hernia and dumping syndrome appears to be non-inferior to RYGB and possible superior. Within the follow-up period, no patients experienced a bowel obstruction secondary to internal herniation and there were no patients with symptoms of dumping syndrome. Up to 40% of patients with RYGB can experience symptoms of dumping syndrome ranging from a mild severe [15]. Although it is likely that recall bias and observer bias contribute to the low numbers in our cohort, it has been shown in the literature that dumping syndrome may be less common in patients undergoing OAGB [16]. Internal herniation occurs in up to 9–10% of patients undergoing RYGB [17]; we had no patients in our cohort with internal hernia and this appears in line with published data showing significantly lower rates of internal herniation related to OAGB [18]. Internal hernia can have significant sequelae, typically requiring emergent surgical intervention, possibly contributing to chronic vague symptoms in these patients [19].

RYGB is an excellent procedure; however, the side effect profile should give surgeons pause—the increased rate of internal herniation, the increased risk of dumping syndrome can significantly affect patient’s quality of life. Even with closure of mesenteric windows in the setting of bariatric metabolic surgery when patients lose weight, this space may open again. Chronic unexplained abdominal pain attributable to RYGB also exists in 15–30% of patients [19]; it is unclear exactly why this occurs but the decrease rate in OAGB leads to the hypothesis that the entero-enterostomy may play a role [18].

There are limitations in our study of note. Our cohort has a selection bias towards high BMI and high rate of associated medical problems as these patients are those that typically benefit from an intestinal diversion procedure. There is likely confounding that exists given patients had differing BP limb lengths tailored to them specifically. The retrospective nature of interpretation of the data as well as missing or incorrectly recorded data sets makes it inappropriate to make concrete conclusions based on the results that we have presented; a lack of appropriate comparator/control group also makes it difficult to make inference with regards to statistical significance. We have opted to overcome these limitations by describing our data in full transparency of these limitations and drawing upon correlations we have noted in line with the published literature and in future work be able to overcome these issues.

## Conclusion

In this, the largest Australasian series to date, we have found our OAGB populations outcomes to be in line with published literature with the possibility that results may be superior in the hands of high-volume surgeons. The prevalence of bile reflux is overall low and no current published evidence exists to support an increased risk of metaplasia or malignancy related to bile within the stomach. There is convincing evidence that the complication profile of OAGB is significantly less than RYGB, particularly with respect to internal herniation and dumping syndrome.

Further research is required to comprehensively assess long term outcomes with respect to long-term durability of weight loss and impact on associated medical problems as well as whether any significant nutritional deficits are more or less likely to occur compared to RYGB. The weight loss, control of associated medical problems and utility in both primary and revisional settings suggest that OAGB is an excellent option for the appropriate surgical candidate.

## Data Availability

The data that support the findings of this study are available from the corresponding author, DT, upon reasonable request.
